# TRAIL Deficiency Contributes to Diabetic Nephropathy in Fat-Fed ApoE^-/-^ Mice

**DOI:** 10.1371/journal.pone.0092952

**Published:** 2014-03-25

**Authors:** Siân P. Cartland, Jonathan H. Erlich, Mary M. Kavurma

**Affiliations:** 1 Centre for Vascular Research, University of New South Wales, Sydney, New South Wales, Australia; 2 Prince of Wales Clinical School, University of New South Wales, Sydney, New South Wales, Australia; 3 The Heart Research Institute, Sydney, New South Wales, Australia; 4 Faculty of Medicine, The University of Sydney, Sydney, New South Wales, Australia; Nazarbayev University, Kazakhstan

## Abstract

**Background:**

We recently demonstrated that TNF-related apoptosis-inducing ligand (TRAIL) is protective of diet-induced diabetes in mice. While TRAIL has been implicated in chronic kidney disease, its role *in vivo* in diabetic nephropathy is not clear. The present study investigated the role of TRAIL in the pathogenesis of diabetic nephropathy using TRAIL^-/-^ApoE^-/-^ mice.

**Methods:**

TRAIL^-/-^ApoE^-/-^ and ApoE^-/-^ mice were fed a high fat diet for 20 w. Plasma glucose and insulin levels were assessed over 0, 5, 8 and 20 w. At 20 w, markers of kidney function including creatinine, phosphate, calcium and cystatin C were measured. Changes in mRNA expression of MMPs, TIMP-1, IL-1β and IL-18 were assessed in the kidney. Functional and histological changes in kidneys were examined. Glucose and insulin tolerance tests were performed.

**Results:**

TRAIL^-/-^ApoE^-/-^ mice had significantly increased urine protein, urine protein:creatinine ratio, plasma phosphorous, and plasma cystatin C, with accelerated nephropathy. Histologically, increased extracellular matrix, mesangial expansion and mesangial cell proliferation in the glomeruli were observed. Moreover, TRAIL^-/-^ApoE^-/-^ kidneys displayed loss of the brush border and disorganisation of tubular epithelium, with increased fibrosis. TRAIL-deficient kidneys also had increased expression of MMPs, TIMP-1, PAI-1, IL-1β and IL-18, markers of renal injury and inflammation. Compared with ApoE^-/-^ mice, TRAIL^-/-^ApoE^-/-^ mice displayed insulin resistance and type-2 diabetic features with reduced renal insulin-receptor expression.

**Conclusions:**

Here, we show that TRAIL-deficiency in ApoE^-/-^ mice exacerbates nephropathy and insulin resistance. Understanding TRAIL signalling in kidney disease and diabetes, may therefore lead to novel strategies for the treatment of diabetic nephropathy.

## Introduction

The leading cause of end-stage renal disease, which can result in disability and mortality of diabetic patients, is diabetic nephropathy (DN) [1]. Multiple mechanisms contribute to the progression of this disease, including haemodynamic pathways, hyperglycemia, hyperlipidemia, oxidative stress, inflammatory cytokines as well as genetic disposition [2]. The pathophysiology of DN is complex and not completely understood.

TNF-related apoptosis-inducing ligand (TRAIL) is a TNF superfamily cytokine that can promote apoptosis, necroptosis [3] and survival of cells, displaying pleiotropic functions both *in vitro* and *in vivo* (reviewed in [4]–[7]). This is not surprising; TRAIL displays complex signalling with the ability to bind 5 different receptors in humans. Four of these receptors are membrane-bound; namely death receptor-4, and -5, as well as decoy receptors-1, and -2. TRAIL also binds the soluble receptor, osteoprotegerin known to regulate osteoclastogenesis.

Recent studies have suggested a possible role for TRAIL in the pathogenesis of DN. In normal kidneys, TRAIL expression is localised in the tubules, but not the glomeruli [8]. Transcriptomic and bioinformatic studies in human diabetic kidneys, showed increased expression of TRAIL, correlating with severity of renal disease [9]. Moreover, TRAIL protein expression in kidney tissue sections was associated with tubular atrophy, interstitial fibrosis and inflammation [8]. These studies suggest that increased expression of TRAIL in a diabetic milieu may play an apoptotic role and modulate kidney injury in DN.

We have previously demonstrated that in response to a high-fat diet (HFD) for 12 w, TRAIL gene deletion in ApoE^-/-^ mice resulted in increased systemic inflammation, diabetes and accelerated atherosclerosis [10]. Chronic kidney disease and DN is associated with accelerated atherosclerosis. Interestingly, circulating levels of soluble TRAIL are considered a negative marker for inflammation, inversely associated with the mortality risk in chronic kidney disease patients [11]. Circulating TRAIL levels are also decreased in the sera of haemodialysis patients [12]. In contrast to the observed tissue expression of TRAIL in DN [8], [9], the reduced circulating TRAIL levels implicate a protective role for TRAIL in chronic kidney disease.

To date, no kidney phenotype has been described in TRAIL-deficient mice. Thus, in this study we aimed to identify whether TRAIL plays a progressive or protective role in DN. We examined the effects of a 20 w HFD on TRAIL^-/-^ApoE^-/-^ mice and ApoE^-/-^ mice. Here, TRAIL-deficient mice displayed increased renal pathology as well as type-2 diabetes. Understanding TRAIL signalling in diabetic nephropathy may therefore offer new strategies for the treatment of diabetes and renal diseases.

## Materials and Methods

### Mice

Male TRAIL^-/-^ApoE^-/-^ and ApoE^-/-^ mice were used for all studies [10]. Six week old mice were placed on a HFD (Specialty Feeds; Perth, Australia) for 20 w in specific pathogen-free conditions with 12∶12 h light-dark cycles, and free access to water and food. To minimise stress, mice were monitored daily and handled frequently. Body weights were measured and blood was sampled via tail vein throughout the study. At 18 and 19 w into the HFD, glucose (overnight fasted) and insulin (non-fasted) tolerance tests were performed. Either D-Glucose (1 g/kg body weight; Sigma-Aldrich, Sydney, Australia) or 1 U/kg of body weight of human insulin (Roche, Sydney, Australia) was injected into mice intraperitoneally, followed by plasma glucose measurements over 2 h using a glucometer (Accuchek Performa, Roche, Sydney, Australia) [10]. At the end of the diet period, and after overnight fasting, mice were anaesthetised by intraperitoneal injection of ketamine (100 mg/kg) and xylazine (10 mg/kg), and culled by cardiac exsanguination. Kidneys were quickly harvested, weighed and fixed in 10% formaldehyde for immunohistochemistry (IHC) analyses or snap frozen for expression studies.

### Ethics statement

All animals were handled according to the Animal Care and Ethics Committee (ACEC) guidelines at UNSW (Sydney, Australia); the protocol was approved by the ACEC of UNSW with Ethics approval number 11/71B.

### Food intake

Pre-weighed food was placed into clean cages at the beginning of the week. At the end of the week, the unconsumed food was collected and weighed. This amount was subtracted from the given amount. Daily food intake was calculated; when there was more than one mouse housed per cage, the food intake was averaged over the number of mice in the cage. Food intake was measured over the final 4 weeks of HFD.

### Plasma and Urine analyses

At 20 w, urine was obtained for measurement of protein (Pierce, Rockford, U.S.A), calcium (Cayman Chemical, An Arbour, MI, USA) and creatinine (Abcam, Cambridge). Plasma obtained at time of sacrifice was stored at -80°C in EDTA-NA_2_ or heparin tubes. Plasma protein (Pierce, Rockford, U.S.A), creatinine (Abcam, Cambridge, UK), calcium (Cayman Chemical, An Arbour, MI, USA), phosphorus (VetScan; Abaxis, Union City, CA, USA), cystatin C (R & D Systems), glucose (Cayman Chemical, An Arbour, MI, USA) and insulin (Mercodia, Uppsala, Sweden) were subsequently assessed.

### IHC and light microscopy

Fixed kidneys embedded in paraffin were used in subsequent IHC. Tissue architecture was assessed following hematoxylin and eosin staining. Kidney sections were stained with Periodic acid Schiff (PAS), alizarin red, F4/80 (macrophage; 1∶50; AbD serotec; Oxford, UK); Collagen IV (1∶500; Abcam, Cambridge, UK), Vimentin (1∶500; Abcam, Cambridge, UK) and Masson's Trichrome. Sections were examined to assess the effect of TRAIL-deficiency in ApoE^-/-^ kidneys with particular emphasis on tubular cells, mesangial expansion in glomeruli and infiltration of macrophages. All IgG controls were negative. Digital images were captured using a BX53 or DP72 microscope (Olympus). For quantification of staining in tissues, positive staining was determined using cellSens imaging software (Olympus). For kidneys, 12–17 random viewing fields (inner and outer cortex), and approximately 20–25 glomeruli were assessed per mouse. Thresholds for positive staining were determined for each antibody; sections were analysed by an investigator blinded to mouse genotype.

### RNA extraction and quantitative real time polymerase chain reaction (PCR)

Kidneys snap frozen in liquid N_2_ were stored at −80°C until use. Tissues were homogenised (MP Biomedicals: Sydney, Australia) and RNA was extracted using the Qiagen all prep DNA/RNA/protein kit. RNA was reverse transcribed as previously described using iScript (BioRad: Sydney, Australia) [10]. Quantitative real-time PCR was performed using SensiFast (Bioline, Sydney, Australia) and the Rotor-Gene 6000 (Corbett Research, Qiagen; Melbourne, Australia). Quadruplicate samples were run, and relative changes in gene expression between TRAIL^-/-^ApoE^-/-^ and ApoE^-/-^ kidneys were determined using the 2-ΔΔcT method qPCR [10]. Values were normalised to the housekeeping gene β-actin. Primer sequences for each gene examined can be found in Table 1.

**Table 1:** Murine Primer Sequences.

Gene	Forward Primer 5′ to 3′	Reverse Primer 5′ to 3′
Fibronectin	ACAGAAATGACCATTGAAGG	TGTCTGGAGAAAGGTTGATT
PAI-1	TCTGGGAAAGGGTTCACTTTACC	GACACGCCATAGGGAGAGAAG
TIMP-1	CGAGACCACCTTATACCAGCG	ATGACTGGGGTGTAGGCGTA
MMP-2	CAGGGCACCTCCTACAACAG	CAGTGGACATAGCGGTCTCG
MMP-9	CCAAGGGTACAGCCTGTTCCT	GCACGCTGGAATGACTAAGC
IL-1β	GTTTCTGCTTTCACCACTCCA	GAGTCCAATTTACTCCAGGTCAG
IL-18	GACTCTTGCGTCAACTTCAAGG	CAGGCTGTCTTTTGTCAACGA
Osteopontin	CCCGGTGAAAGTGACTGATTC	ATGGCTTTCATTGGAATTGC
PPAR-γ	CACAATGCCATCAGGTTTGG	GCTGGTCGATATCACTGGAGATC
TNF-α	CAGGCGGTGCCTATGTCTC	CGATCACCCCGAAGTTCAGTAG
GLUT2	GGCTAATTTCAGGACTGGTT	TTTCTTTGCCCTGACTTCCT
GLUT4	GTGACTGGAACACTGGTCCTA	CCAGCCACGTTGCATTGTAG
Insulin Receptor	TTTGTCATGGATGGAGGCTA	CCTCATCTTGGGGTTGAACT
β-actin	AACCGTGAAAAGATGACCCAGAT	CACAGCCTGGATGGCTACGTA

### Statistics

For data analysis, GraphPad Prism version 6.0 (GraphPad Software, San Diego, CA, USA) was used. Unless stated, statistical comparisons were performed using a Mann-Whitney *t*-test and one-way ANOVA, where appropriate. Results are expressed as mean ± SEM; *p*<0.05 was considered significant.

## Results

### 20 w HFD fed TRAIL^-/-^ApoE^-/-^ mice have increased markers of renal injury

TRAIL^-/-^ApoE^-/-^ mice at 20 w exhibited markedly increased plasma phosphorus, plasma cystatin C, urine calcium, urine protein (Table 2) and urine protein:creatinine ratio compared to ApoE^-/-^ mice (62.32±8.81 mg/mmol vs. 31.94±4.90 mg/mmol; p<0.05). Furthermore, TRAIL^-/-^ApoE^-/-^ mice had significantly reduced plasma protein (Table 2). In contrast, there was no change in wet kidney weights, plasma or urine creatinine levels (Table 2). Calcification in kidneys is another marker of renal injury and chronic kidney failure. Histologically, we observed significantly elevated alizarin red staining in TRAIL^-/-^ApoE^-/-^ vs ApoE^-/-^ kidneys (Figure S1A). These studies suggest that a deficiency of TRAIL in ApoE^-/-^ mice results in significant increases in plasma and urine markers indicative of renal injury.

**Table 2:** Organ weights, blood and urine measurements from ApoE^-/-^ and TRAIL^-/-^ApoE^-/-^ mice after 20 w HFD.

	*ApoE^-/-^*	*TRAIL^-/-^ApoE^-/-^*
Kidney weight (g)	0.22±0.01	0.22±0.01
Plasma Calcium (mmol/L)	2.66±0.29	2.38±0.21
Plasma Phosphorus (mmol/L)	2.47±0.22	3.51±0.29*
Plasma Creatinine (μmol/L)	443.1±50.02	366.7±34.37
Plasma Cystatin C (mg/L)	407.43±42.74	561.14±41.27*
Plasma Protein (g/L)	64.3±3.76	53.0±2.05*
Urine Calcium (mmol/L)	1.48±0.63	2.98±0.55
Urine Creatinine (μmol/L)	947.8±57.62	1057.0±60.37
Urine Protein (g/L)	30.79±5.50	62.76±7.22*

Measurements are from n = 7–11 mice per group. Results are expressed as mean ± SEM, *p< 0.05 and **p<0. 01, by Mann-Whitney U test.

### 20 w HFD fed TRAIL^-/-^ApoE^-/-^ kidneys display nephropathy

**Figure 1:**
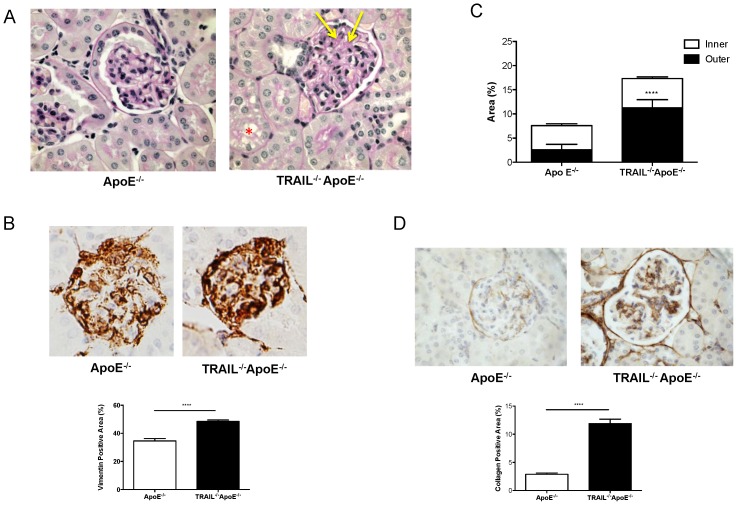
TRAIL^-/-^ApoE^-/-^ mice have increased renal injury. Representative sections (40X magnification) of mouse kidney after 20 w of HFD stained with (a) Periodic acid Schiff showing relative increased mesangial matrix (yellow arrows) and increased cellularity in mesangial regions with local areas of tubular degeneration and loss of brush boarder (red asterix), (b) vimentin demonstrating increased matrix, (c) collagen IV (total kidney) showing increased collagen staining throughout the interstitium and (d) glomeruli. Stains were quantified as described in the Methods. Measurements are from n = 5 mice/genotype, 20–25 glomeruli per mouse (vimentin and collagen IV), or 11–15 images each of inner and outer cortex for collagen IV. Results are expressed as mean ± SEM, ****p<0.0001 by Mann-Whitney U test.

Both ApoE^-/-^ and TRAIL^-/-^ApoE^-/-^ mice developed abnormal glomerular histology. TRAIL^-/-^ApoE^-/-^ kidneys however, had more segmental glomerular matrix accumulation and segmental cell proliferation as demonstrated by PAS staining (Figure 1A). Increased mesangial cell proliferation in TRAIL^-/-^ApoE^-/-^ glomeruli was confirmed with vimentin, a marker of mesangial cells (Figure 1B). Not only did TRAIL^-/-^ApoE^-/-^ tubular cells display increased loss of brush border and disorganization of the tubular epithelium, as well as some cell shedding, they also showed vacuolization, more so than ApoE^-/-^ tubular cells (Figure 1A). Furthermore, TRAIL^-/-^ApoE^-/-^ kidneys demonstrated greater fibrosis as evident by collagen IV staining, particularly in the outer cortex (Figure 1C), and also in the glomeruli (Figure 1D). Consistent with this, TRAIL^-/-^ApoE^-/-^ kidneys displayed increased collagen expression by Masson's trichrome (Figure S1B). These studies suggest that TRAIL deficiency contributes to the greater nephropathy seen in HFD-fed ApoE^-/-^ mice.

### Expression of markers of fibrosis and renal disease

**Figure 2:**
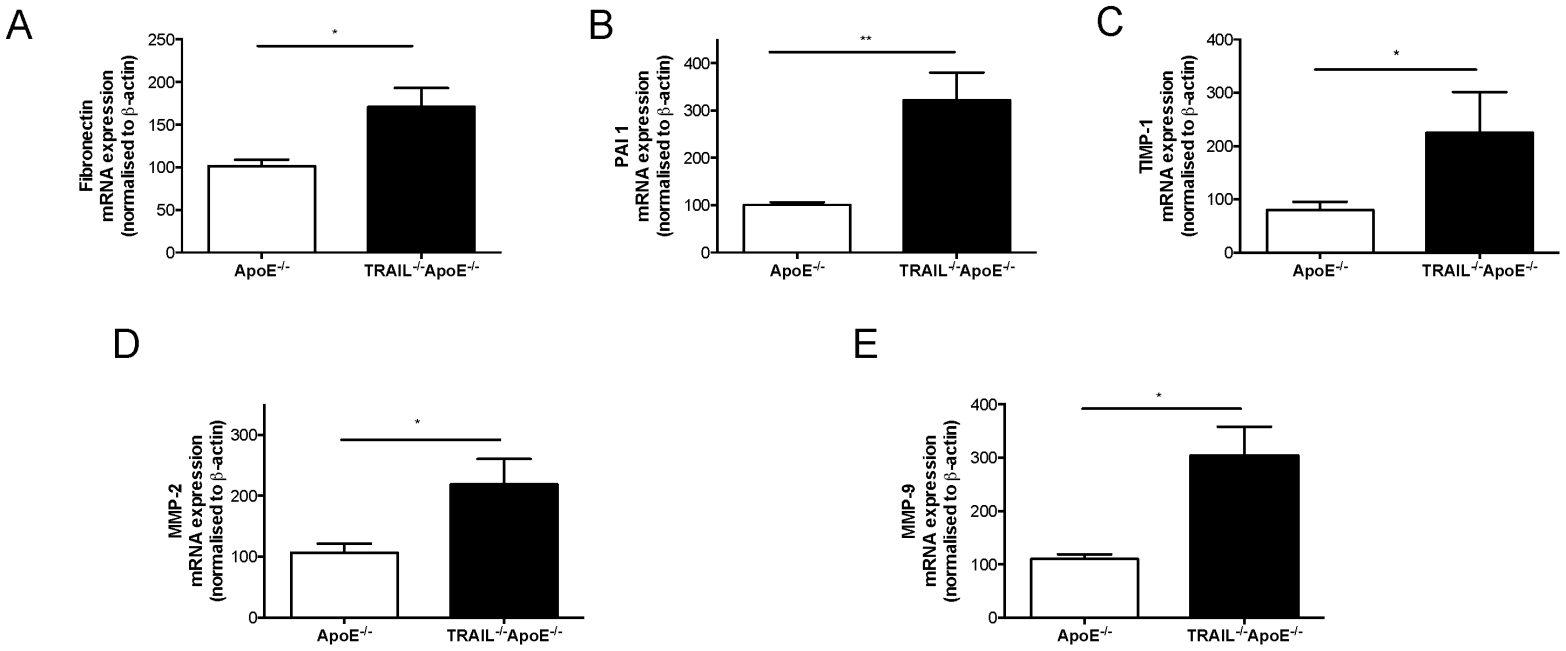
Kidneys from TRAIL^-/-^ApoE^-/-^ mice have increased expression of genes indicative of fibrosis. mRNA expression for (a) fibronectin, (b) PAI-1, (c) TIMP-1, (d) MMP-2 and (e) MMP-9 from kidneys. All levels were normalized to β-actin; n = 6/ genotype. Results are expressed as mean ± SEM, *p<0.05 and **p<0.01 by Mann-Whitney U test.

Renal disease due to fibrosis is caused in part, by an impairment of proteolytic factors that regulate extracellular matrix throughput. Excess accumulation of fibronectin (a key matrix protein) for example, is a common feature in human renal disease [13]. Importantly, TRAIL^-/-^ApoE^-/-^ kidneys had elevated mRNA expression of fibronectin (Figure 2A). Proteases such as plasminogen activator inhibitor-1 (PAI-1) and matrix metalloproteinases (MMPs) including tissue inhibitor of metalloproteinases-1 (TIMP-1), MMP-2 and -9 have also been implicated in fibrosis and renal injury [14]. PAI-1, TIMP-1, as well as MMP-2 and -9 were significantly increased in the kidneys of TRAIL^-/-^ApoE^-/-^ mice (Figures 2B-E). These findings demonstrate that TRAIL-deficient kidneys have altered expression of genes regulating extracellular matrix turnover and fibrosis.

### Increased expression of pro-inflammatory cytokines in TRAIL^-/-^ApoE^-/-^ kidneys

**Figure 3:**
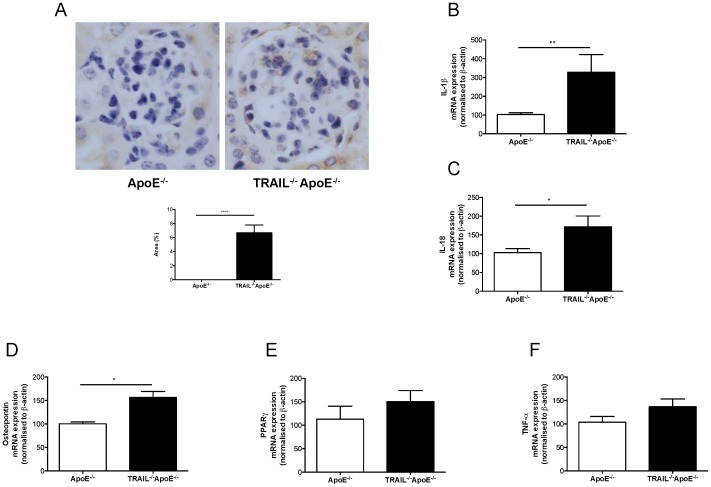
TRAIL^-/-^ApoE^-/-^ mice have increased macrophage infiltration and genes of inflammation. Representative sections (40X magnification) of mouse kidney after 20 w HFD stained for the pan macrophage marker (a) F4/80. Kidney mRNA expression for (b) IL-1β, (c) IL-18, (d) osteopontin (e) PPAR-γ and (f) TNF-α. All levels were normalized to β-actin; n = 5-6/genotype. Results expressed as mean ± SEM, *p<0.05, **p<0.01 and ****p<0.0001 by Mann-Whitney U test.

In addition to fibrosis, nephropathy is associated with increased inflammation. Indeed 20 w HFD-fed TRAIL^-/-^ApoE^-/-^ mice had significantly elevated white blood counts (12.48×10^3^/μl ±1.33 vs. 4.76×10^3^/μl ±0.69; p<0.01) and increased spleen weights (0.19 g ±0.01 vs. 0.14 g±0.01; p<0.01). Histologically, significantly increased macrophage infiltration as assessed by F4/80 positive staining was observed in the glomeruli of TRAIL^-/-^ApoE^-/-^ vs ApoE^-/-^ mice (Figure 3A). Furthermore, TRAIL^-/-^ApoE^-/-^ kidneys displayed increased mRNA expression of pro-inflammatory cytokines IL-1βIL-18 (Figures 3B-C), and their downstream target gene, osteopontin [15], [16] (Figure 3D), a macrophage chemotactic and adhesion molecule. While an increase for PPAR-γ and TNF-αexpression was seen in TRAIL^-/-^ApoE^-/-^ kidneys (Figures 3E-F), this elevation however was not significant. Thus, TRAIL is protective of systemic and kidney-specific inflammation in HFD-fed ApoE^-/-^ mice.

### TRAIL^-/-^ApoE^-/-^ mice display features of type-2 diabetes and altered insulin signalling

**Figure 4:**
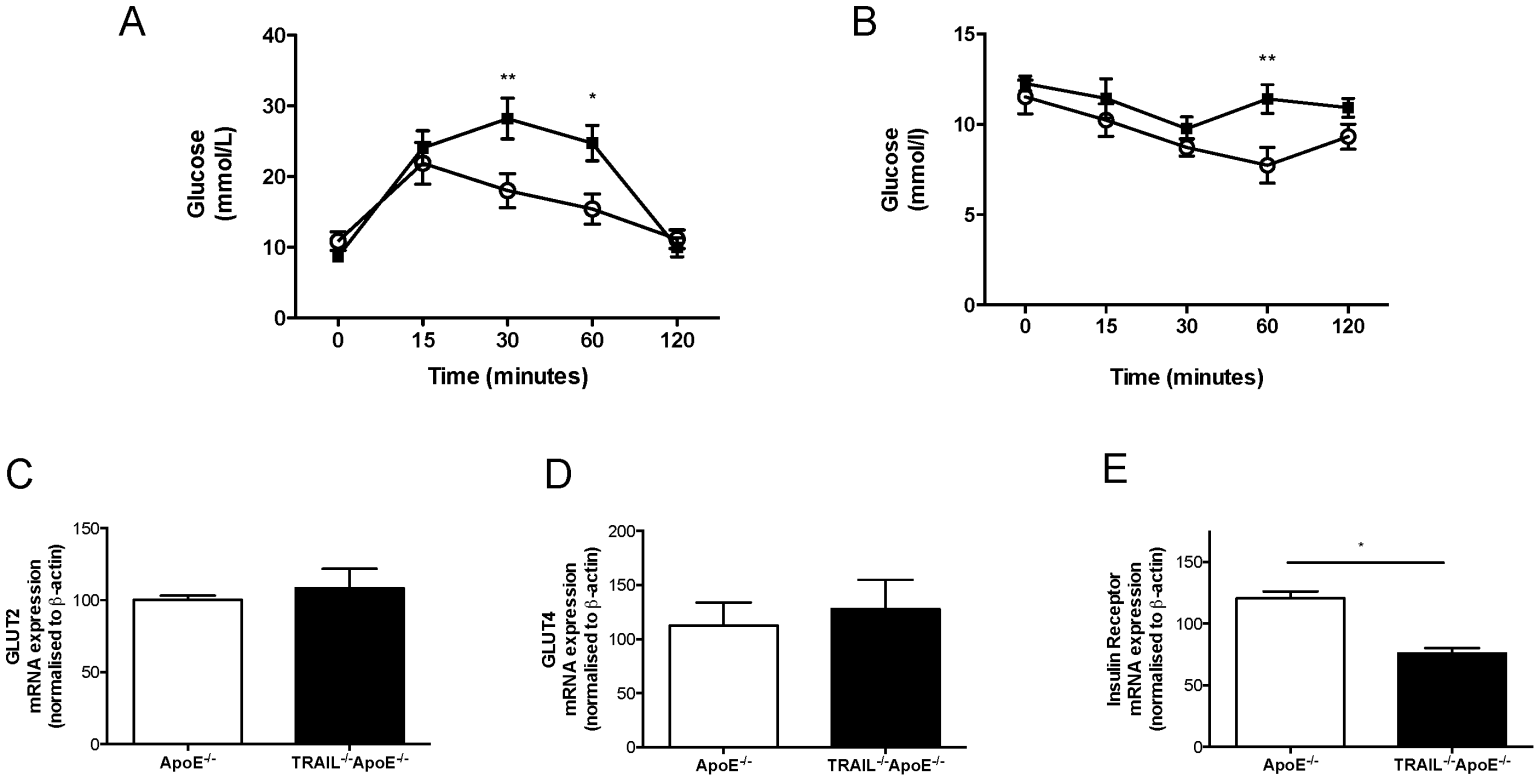
TRAIL^-/-^ApoE^-/-^ mice show impaired glucose and insulin tolerance and kidneys have altered insulin signalling. (a) Glucose and (b) insulin tolerance tests of ApoE^-/-^ (open circles) and TRAIL^-/-^ApoE^-/-^ (▪) mice (n = 6–11/genotype). mRNA levels for (c) GLUT2, (d) GLUT4 and (e) insulin receptor were measured in the kidney. All mRNA levels were normalized to β-actin; n = 5-6/genotype. Results expressed as mean ± SEM, *p<0.05, **p<0.01, ***p<0.001 and ****p<0.0001 by Mann-Whitney U test and ANOVA.

HFD treatment caused significant increases in body weight and plasma glucose levels at 8 w in TRAIL^-/-^ApoE^-/-^ mice, consistent with the development of diet-induced diabetes. Strikingly at 20 w, TRAIL^-/-^ApoE^-/-^ mice had significantly reduced plasma glucose and insulin levels with no change in body weight compared to ApoE^-/-^ mice (Table 3). Interestingly, food intake was significantly reduced in TRAIL^-/-^ApoE^-/-^ mice (Figure S2), even though prior to sacrifice, these mice still displayed impaired glucose clearance (Figure 4A) and delayed insulin sensitivity compared to ApoE^-/-^ mice, with insulin intolerance observed at 60 min (Figure 4B).

**Table 3:** Body weights, plasma glucose and insulin levels in HFD-fed ApoE^-/-^ and TRAIL^-/-^ApoE^-/-^ mice.

	Body weight (g)		Plasma Glucose (mmol/L)		Plasma Insulin (pmol/L)	
Weeks of HFD	*ApoE^-/-^*	*TRAIL^-/-^ApoE^-/-^*	*ApoE^-/-^*	*TRAIL^-/-^* *ApoE^-/-^*	*ApoE^-/-^*	*TRAIL^-/-^ApoE^-/-^*
0	20.90±0.84	19.37±0.89	10.74±1.57	11.47±0.76	24.1 9±4.26	20.41±4.42
5	28.15±0.88	27.28±0.75	12.90±1.40	13.00±1.23	29.64±6.73	30.39±8.46
8	28.44±0.65	30.77±0.73*	15.20±0.83	18.04±0.78*	35.39±9.73	28.31±7.17
20	31.24±1.95	30.08±0.76	18.75±1.33	8.65±1.18****	33.31±6.22	19.06±3.08*

Measurements are from n = 5–11 mice per group. Results are expressed as mean ± SEM, *p<0.05 and ****p<0. 0001, by Mann-Whitney U test.

Insulin signalling is critical for kidney function. We therefore assessed the expression of the insulin receptor, and glucose transporters (GLUT-2 and GLUT-4), important in glucose metabolism in the kidney. No significant differences in GLUT-2 or -4 mRNA were observed in kidneys of TRAIL^-/-^ApoE^-/-^ vs. ApoE^-/-^ mice (Figures 4C-D). In contrast, insulin receptor expression was significantly reduced in TRAIL-deficient kidneys (Figure 4E). These findings suggest that HFD-fed TRAIL^-/-^ApoE^-/-^ mice are glucose and insulin intolerant, and that kidneys of TRAIL-deficient mice have altered expression of insulin signalling molecules.

## Discussion

DN is a condition observed in >40% of diabetic patients in the US (American Diabetes Association). It is a condition characterized by thickening of basement membranes within glomeruli and tubules, as well as mesangial expansion and cell proliferation, increased production of matrix factors, and tubulointerstitial fibrosis [2]. A direct role for TRAIL in DN has not been established. However, there is growing interest supporting its association with diabetes and renal diseases. For example, increased TRAIL expression, apoptosis and scarring have been observed in kidneys of DN patients [8]. In cell culture experiments using proximal tubular cells, a combination of high glucose, TNF-αand IFN-γ increased TRAIL expression, which correlated with modest increases in apoptosis [8]. However, the role of glucose alone on TRAIL expression in these cells was not examined [8]. These studies suggest that increases in TRAIL expression, together with a pro-inflammatory milieu may augment apoptosis and disease progression. In support, circulating TRAIL levels appear to be elevated in diabetic patients with micro- and macroalbuminuria; however, this finding failed to achieve significance after multiple variant regression analysis [17]. In contrast, circulating TRAIL levels are reduced in patients with chronic kidney disease [11] and in patients undergoing haemodialysis [12]. Chronic kidney disease is a complication of heart transplantation, and heart transplant recipients displayed lower serum TRAIL levels, associated with a decline in glomerular filtration rate [18]. These studies suggest that TRAIL may be protective of kidney disease by inhibiting apoptosis. TRAIL's role *in vivo* is therefore conflicting and controversial. As such, we need to have a firm understanding of how TRAIL functions in the kidney in order to better develop TRAIL-dependent rational and safe interventions.

In the present study, we show the first demonstration that TRAIL-gene deletion in HFD-fed ApoE^-/-^ mice results in accelerated nephropathy and insulin resistance. We have revealed that TRAIL^-/-^ApoE^-/-^ mice in response to a HFD have increased plasma phosphorus, urine protein and urine protein:creatinine ratio. No significant changes in urine or plasma creatinine levels between genotype were observed. In contrast, plasma cystatin C levels, another biomarker of kidney function, were significantly elevated in the TRAIL-deficient mice. Compared to creatinine, cystatin C has a smaller volume of distribution, and levels are less dependent on muscle mass and other factors, potentially reflecting a better marker of glomerular filtration rate than serum creatinine [19]. Never the less, collectively, these changes observed in TRAIL-deficient mice are indicative of renal injury and kidney failure. Consistent with these findings, TRAIL^-/-^ApoE^-/-^ mice had significantly exacerbated renal pathology, associated with mesangial expansion, fibrosis and inflammation. Furthermore, TRAIL-deficient kidneys displayed increases in extracellular matrix modulating genes.

TRAIL deficiency has previously been associated with accelerated autoimmune type I diabetes [20]. In this report, TRAIL^-/-^ApoE^-/-^ mice had significantly elevated weight gain and hyperglycaemia at 8 w, and surprisingly, these parameters were not sustained by 20 w. TRAIL^-/-^ApoE^-/-^ mice also displayed reduced plasma insulin levels at 8 and 20 w. However, these mice had impaired insulin tolerance, suggesting a type-2 diabetic phenotype. Insulin resistance is a condition when the body's cells fail to respond to insulin, regardless of the levels of insulin. Notably, TRAIL^-/-^ApoE^-/-^ mice had significantly reduced insulin receptor expression in kidneys; a finding that may explain the impaired insulin sensitivity observed in TRAIL^-/-^ApoE^-/-^ mice at 60 min. Here we show that TRAIL-deficiency is also associated with insulin resistance in response to a HFD.

Reduced appetite is a common problem in inflammatory conditions including advanced kidney disease. Consistent with more severe nephropathy, TRAIL^-/-^ApoE^-/-^ mice had reduced appetite and were eating less than ApoE^-/-^ mice within the last 4 w of the study; supported by the markedly reduced plasma glucose levels at 20 w, even though TRAIL-deficient mice were still insulin resistant. Notably, severe renal dysfunction is associated with insulin resistance [21]. Furthermore, these mice developed more severe atherosclerosis (data not shown). Other autoimmune disorders such as hyperthyroidism may also play a role in the reduced food intake observed in the TRAIL-deficient mice. However, this requires further investigation.

Inflammation is fundamental in promoting the development and severity of DN. Interestingly, cystatin C levels may also reflect inflammation in CVD or related pathologies [22], [23], and given we observed increased cystatin C levels in TRAIL-deficient mice, suggests a pro-inflammatory environment. The role of cystatin C in inflammation however is controversial. While cystatin C may predict worse outcomes in patients with CVD [24], the relationship between absolute levels of cystatin C and inflammatory cytokines is less clear. For example, despite showing elevated C-reactive protein levels, longitudinal studies of patients measuring cystatin C and renal function following surgery, showed that cystatin C levels were not significantly influenced by inflammation [25]. Thus, the association between cystatin C and inflammation requires further investigation.

Monocytes/macrophages are one of the main cell types in the kidney mediating inflammation and activated macrophages can secrete pro-inflammatory and pro-fibrotic factors including IL-1, IL-18, PAI-1 and MMPs which can result in apoptosis, but also hypercellularity (reviewed in [26]). Our results indicate that TRAIL^-/-^ApoE^-/-^ kidneys have significantly increased F4/80^+^ staining in the glomeruli, indicating increased infiltration of macrophages. TRAIL^-/-^ApoE^-/-^ kidneys display elevated IL-1βIL-18 and osteopontin mRNA expression. Moreover, expression of matrix modulating genes including fibronectin observed in TRAIL-deficient kidneys was significantly increased. This is of importance since our findings suggest that a deficiency in TRAIL leads to a pro-inflammatory state in the kidney, with increased macrophage infiltration, promoting tissue injury, cell expansion, fibrosis and scarring.

TRAIL was identified as a member of the TNF ligand family almost 20 years ago, capable of inducing programmed cell death [27], [28]. It is expressed on all tissues, including the kidney [29], without the induction of apoptosis, suggesting that TRAIL's cytotoxic role *in vivo* is selective. Recent studies also implicate TRAIL in regulating necroptosis [3], or programmed necrosis, independent of caspase activation. While we did not assess apoptosis or necroptosis in the kidney, our previous findings suggest that increased macrophage accumulation in TRAIL^-/-^ApoE^-/-^ mice is associated with active caspase-3-positive cells, not only atherosclerotic lesions, but also in pancreatic islets [10]. Interestingly, in the streptozotocin rat diabetic model, renal TRAIL expression was increased at 16 w, with TRAIL expression further augmented in rats treated with valsartan and/or mycophenolate mofetil [30], agents which inhibit renal apoptosis and protect kidney function [31]. Thus, TRAIL may play different roles on different cells and in varying circumstances. Our data suggests that TRAIL may be important for mesagial cell turnover, and for clearing inflammatory cells, such as macrophages in the kidney. This is supported by our previous work suggesting that TRAIL-deficiency leads to increased systemic inflammation and apoptosis, to exacerbate diabetes and atherosclerosis [10].

TRAIL signalling is complicated; amplified with the identification of alternatively spliced variants [32], [33], and multifaceted mechanisms involving 5 receptors in humans. TRAIL also has the ability to promote cell survival, proliferation and differentiation via activation of NFκB, mitogen-activated protein kinase (MAPK), c-Jun N-terminal kinase (JNK) and phosphatidylinositide 3-kinase (PI3K)-dependent pathways (reviewed in [6], [7]). Thus, TRAIL is a control switch, and depending on the environment e.g. expression of its receptors, TRAIL concentration, inflammatory milieu and specific cell types, it can decide whether a cell dies or survives. Notably, all TRAIL receptors, except decoy receptor-2, are expressed in the normal kidney. Osteoprotegerin is the only identified soluble receptor for TRAIL, known to inhibit TRAIL's function(s) and circulating osteoprotegerin levels seem to play a role in disease, particularly in diabetes and diabetic complications including kidney diseases (reviewed in [34]). Importantly, osteoprotegerin levels are independently associated with the severity of DN [17], and in a transcriptomic study using DN kidney biopsies, osteoprotegerin (together with TRAIL) was one of the genes conferring the highest expression [9]. While tissue expression of osteoprotegrin in TRAIL^-/-^ApoE^-/-^ kidneys needs further investigation, we failed to show a difference in circulating osteoprotegerin levels in these mice [35].

TRAIL can modulate host defence mechanisms, important in controlling tumour growth [36]-[38]. In fact, lymphocytes, monoctyes and dendritic cells, are important in supressing TRAIL-mediated tumour cell growth [39]–[42]. We have previously shown that TRAIL expression is increased following mechanical injury to vascular smooth muscle cells *in vitro*, and after peri-vascular cuff placement to femoral arteries of wild-type mice [43]. Importantly, mechanical injury promoted vascular smooth muscle cell proliferation and survival [43]. Given that TRAIL expression is increased in kidneys of DN patients, it is therefore enticing to speculate that TRAIL may be expressed in damaged or injured tissues as a mechanism of host defence; to modulate levels of inflammation and apoptosis and/or survival in damaged tissues. In this report, we show that a deficiency in TRAIL in ApoE^-/-^ mice resulted in a heightened inflammatory state in the kidney, as well as exacerbated nephropathy. This suggests that TRAIL is indeed protective of inflammation and kidney tissue damage in ApoE^-/-^ mice. Thus, TRAIL plays an important role in attenuating the development of DN. Here, we also illustrate the importance of more studies to delineate the critical and complex function(s) of TRAIL in this setting.

## Supporting Information

Figure S1 **Kidneys from TRAIL^-/-^ApoE^-/-^ mice show increased calcification and collagen.** (a) Calcium was measured by Alizarin Red staining and (b) collagen by Masson's Trichrome. Measurements are from n = 8–11 mice per genotype. Results are expressed as mean ± SEM, *p<0.05 and **p<0.01 by Mann-Whitney U test.
(TIFF)

Figure S2 **TRAIL^-/-^ApoE^-/-^ mice consume less food.** Food intake for ApoE^-/-^ (open circles) and TRAIL^-/-^ApoE^-/-^ (closed squares) mice was measured as described in the Materials and Methods. Measurements are from n = 3 mice/genotype. Linear regression was used to assess significance of slopes for each line; expressed as mean ± SEM, p<0.0001.
(TIFF)

## References

[1] Maisonneuve P, Agodoa L, Gellert R, Stewart JH, Buccianti G, et al (2000) Distribution of primary renal diseases leading to end-stage renal failure in the United States, Europe, and Australia/New Zealand: results from an international comparative study. American Journal of Kidney Diseases: the official journal of the National Kidney Foundation 35: 157–165. 10620560 10.1016/S0272-6386(00)70316-7

[2] Dronavalli S, Duka I, Bakris GL (2008) The pathogenesis of diabetic nephropathy. Nature Clinical Practice Endocrinology & Metabolism 4: 444–452. 10.1038/ncpendmet0894 18607402

[3] Jouan-Lanhouet S, Arshad MI, Piquet-Pellorce C, Martin-Chouly C, Le Moigne-Muller G, et al (2012) TRAIL induces necroptosis involving RIPK1/RIPK3-dependent PARP-1 activation. Cell Death Differ 19: 2003–2014. 22814620 10.1038/cdd.2012.90 PMC3504714

[4] Azahri NS, Kavurma MM (2013) Transcriptional regulation of tumour necrosis factor-related apoptosis-inducing ligand. Cell Mol Life Sci 70: 3617–3629. 23329170 10.1007/s00018-013-1264-x PMC11113472

[5] Di Pietro R, Zauli G (2004) Emerging non-apoptotic functions of tumor necrosis factor-related apoptosis-inducing ligand (TRAIL)/Apo2L. J Cell Physiol 201: 331–340. 15389537 10.1002/jcp.20099

[6] Kavurma MM, Bennett MR (2008) Expression, regulation and function of trail in atherosclerosis. Biochem Pharmacol 75: 1441–1450. 18061141 10.1016/j.bcp.2007.10.020

[7] Kavurma MM, Tan NY, Bennett MR (2008) Death receptors and their ligands in atherosclerosis. Arterioscler Thromb Vasc Biol 28: 1694–1702. 18669890 10.1161/ATVBAHA.107.155143

[8] Lorz C, Benito-Martin A, Boucherot A, Ucero AC, Rastaldi MP, et al (2008) The death ligand TRAIL in diabetic nephropathy. J Am Soc Nephrol 19: 904–914. 18287563 10.1681/ASN.2007050581 PMC2386716

[9] Benito-Martin A, Ucero AC, Santamaria B, Lorz C, Kretzler M, et al (2009) [Transcriptomics illustrate a deadly TRAIL to diabetic nephropathy]. Nefrologia: publicacion oficial de la Sociedad Espanola Nefrologia 29: 13–19. 19240767 10.3265/Nefrologia.2009.29.1.13.1.en.full.pdf

[10] Di Bartolo BA, Chan J, Bennett MR, Cartland S, Bao S, et al (2011) TNF-related apoptosis-inducing ligand (TRAIL) protects against diabetes and atherosclerosis in Apoe (-/-) mice. Diabetologia 54: 3157–3167. 21965021 10.1007/s00125-011-2308-0

[11] Liabeuf S, Barreto DV, Barreto FC, Chasseraud M, Brazier M, et al (2010) The circulating soluble TRAIL is a negative marker for inflammation inversely associated with the mortality risk in chronic kidney disease patients. Nephrology, dialysis, transplantation: official publication of the European Dialysis and Transplant Association - European Renal Association 25: 2596–2602. 10.1093/ndt/gfq042 20190248

[12] Chasseraud M, Liabeuf S, Mozar A, Mentaverri R, Brazier M, et al (2011) Tumor necrosis factor-related apoptosis-inducing ligand and vascular calcification. Therapeutic apheresis and dialysis: official peer-reviewed journal of the International Society for Apheresis, the Japanese Society for Apheresis, the Japanese Society for Dialysis Therapy 15: 140–146. 10.1111/j.1744-9987.2010.00886.x 21426505

[13] Van Vliet A, Baelde HJ, Vleming LJ, de Heer E, Bruijn JA (2001) Distribution of fibronectin isoforms in human renal disease. J Pathol 193: 256–262. 11180174 10.1002/1096-9896(2000)9999:9999<::AID-PATH783>3.0.CO;2-P

[14] Eddy AA (1996) Molecular insights into renal interstitial fibrosis. Journal of the American Society of Nephrology: JASN 7: 2495–2508. 8989727 10.1681/ASN.V7122495

[15] Yu XQ, Fan JM, Nikolic-Paterson DJ, Yang N, Mu W, et al (1999) IL-1 up-regulates osteopontin expression in experimental crescentic glomerulonephritis in the rat. Am J Path 154: 833–841. 10079261 10.1016/S0002-9440(10)65330-8 PMC1866418

[16] Yu Q, Vazquez R, Khojeini EV, Patel C, Venkataramani R, et al (2009) IL-18 induction of osteopontin mediates cardiac fibrosis and diastolic dysfunction in mice. Am J Physiol Heart Circ Physiol 297: H76–85. 19429811 10.1152/ajpheart.01285.2008 PMC2711747

[17] Chang YH, Lin KD, He SR, Hsieh MC, Hsiao JY, et al (2011) Serum osteoprotegerin and tumor necrosis factor related apoptosis inducing-ligand (TRAIL) are elevated in type 2 diabetic patients with albuminuria and serum osteoprotegerin is independently associated with the severity of diabetic nephropathy. Metabolism 60: 1064–1069. 21251686 10.1016/j.metabol.2010.11.002

[18] Malyszko J, Przybylowski P, Koc-Zorawska E, Mysliwiec M (2011) Tumor necrosis factor-related apoptosis-inducing ligand is a marker of kidney function and inflammation in heart and kidney transplant recipients. Transplant Proc 43: 1877–1880. 21693293 10.1016/j.transproceed.2011.03.035

[19] Dharnidharka VR, Kwon C, Stevens G (2002) Serum cystatin C is superior to serum creatinine as a marker of kidney function: a meta-analysis. Am J Kidney Dis 40: 221–226. 12148093 10.1053/ajkd.2002.34487

[20] Lamhamedi-Cherradi SE, Zheng S, Tisch RM, Chen YH (2003) Critical roles of tumor necrosis factor-related apoptosis-inducing ligand in type 1 diabetes. Diabetes 52: 2274–2278. 12941766 10.2337/diabetes.52.9.2274

[21] Charlesworth JA, Kriketos AD, Jones JE, Erlich JH, Campbell LV, et al (2005) Insulin resistance and postprandial triglyceride levels in primary renal disease. Metabolism 54: 821–828. 15931621 10.1016/j.metabol.2005.01.028

[22] Okura T, Jotoku M, Irita J, Enomoto D, Nagao T, et al (2010) Association between cystatin C and inflammation in patients with essential hypertension. Clin Exp Nephrol 14: 584–588. 20809110 10.1007/s10157-010-0334-8

[23] Qing X, Furong W, Yunxia L, Jian Z, Xuping W, et al (2012) Cystatin C and asymptomatic coronary artery disease in patients with metabolic syndrome and normal glomerular filtration rate. Cardiovasc Diabetol 11: 108. 22978689 10.1186/1475-2840-11-108 PMC3473246

[24] Angelidis C, Deftereos S, Giannopoulos G, Anatoliotakis N, Bouras G, et al (2013) Cystatin C: an emerging biomarker in cardiovascular disease. Curr Top Med Chem 13: 164–179. 23470076 10.2174/1568026611313020006

[25] Grubb A, Bjork J, Nyman U, Pollak J, Bengzon J, et al (2011) Cystatin C, a marker for successful aging and glomerular filtration rate, is not influenced by inflammation. Scand J Clin Lab Invest 71: 145–149. 21198422 10.3109/00365513.2010.546879 PMC3072693

[26] Lim AK, Tesch GH (2012) Inflammation in diabetic nephropathy. Mediators of inflammation 2012: 146154. 22969168 10.1155/2012/146154 PMC3432398

[27] Pitti RM, Marsters SA, Ruppert S, Donahue CJ, Moore A, et al (1996) Induction of apoptosis by Apo-2 ligand, a new member of the tumor necrosis factor cytokine family. J Biol Chem 271: 12687–12690. 8663110 10.1074/jbc.271.22.12687

[28] Wiley SR (1995) Schooley K, Smolak PJ, Din WS, Huang CP, et al (1995) Identification and characterization of a new member of the TNF family that induces apoptosis. Immunity 2: 673–682. 10.1016/1074-7613(95)90057-8 8777713

[29] Spierings DC, de Vries EG, Vellenga E, van den Heuvel FA, Koornstra JJ, et al (2004) Tissue distribution of the death ligand TRAIL and its receptors. The journal of histochemistry and cytochemistry: official journal of the Histochemistry Society 52: 821–831. 15150291 10.1369/jhc.3A6112.2004

[30] Chen B, Zhang Y, Liu G, Guan GJ, Hou XH, et al (2008) [Effects of valsartan, mycophenolate mofetil and their combined application on TRAIL and nuclear factor-kappaB expression in the kidneys of diabetic rats]. Zhonghua Yi Xue Za Zhi 88: 540–545. 18649770

[31] Lv W, Zhang Y, Guan G, Li P, Wang J, et al (2013) Mycophenolate mofetil and valsartan inhibit podocyte apoptosis in streptozotocin-induced diabetic rats. Pharmacology 92: 227–234. 24158161 10.1159/000354600

[32] Krieg A, Krieg T, Wenzel M, Schmitt M, Ramp U, et al (2003) TRAIL-beta and TRAIL-gamma: two novel splice variants of the human TNF-related apoptosis-inducing ligand (TRAIL) without apoptotic potential. Br J Cancer 88: 918–927. 12644830 10.1038/sj.bjc.6600772 PMC2377072

[33] Wang P, Lu Y, Li C, Li N, Yu P, et al (2011) Novel transcript variants of TRAIL show different activities in activation of NF-kappaB and apoptosis. Life Sci 89: 839–846. 21952139 10.1016/j.lfs.2011.09.003

[34] Candido R (2014) The osteoprotegerin/tumor necrosis factor related apoptosis-inducing ligand axis in the kidney. Curr Opin Nephrol Hypertens 23: 69–74. 24247823 10.1097/01.mnh.0000437611.42417.7a

[35] Di Bartolo BA, Cartland SP, Harith HH, Bobryshev YV, Schoppet M, et al (2013) TRAIL-Deficiency Accelerates Vascular Calcification in Atherosclerosis via Modulation of RANKL. PLoS One 8: e74211. 24040204 10.1371/journal.pone.0074211 PMC3764101

[36] Cretney E, Takeda K, Yagita H, Glaccum M, Peschon JJ, et al (2002) Increased susceptibility to tumor initiation and metastasis in TNF-related apoptosis-inducing ligand-deficient mice. J Immunol 168: 1356–1361. 11801676 10.4049/jimmunol.168.3.1356

[37] Sedger LM, Glaccum MB, Schuh JC, Kanaly ST, Williamson E, et al (2002) Characterization of the in vivo function of TNF-alpha-related apoptosis-inducing ligand, TRAIL/Apo2L, using TRAIL/Apo2L gene-deficient mice. Eur J Immunol 32: 2246–2254. 12209637 10.1002/1521-4141(200208)32:8<2246::AID-IMMU2246>3.0.CO;2-6

[38] Takeda K, Smyth MJ, Cretney E, Hayakawa Y, Kayagaki N, et al (2002) Critical role for tumor necrosis factor-related apoptosis-inducing ligand in immune surveillance against tumor development. J Exp Med 195: 161–169. 11805143 10.1084/jem.20011171 PMC2193611

[39] Diao Z, Shi J, Zhu J, Yuan H, Ru Q, et al (2013) TRAIL suppresses tumor growth in mice by inducing tumor-infiltrating CD4(+)CD25 (+) Treg apoptosis. Cancer immunology, immunotherapy: CII 62: 653–663. 23143747 10.1007/s00262-012-1370-x PMC11028869

[40] Dorothee G, Vergnon I, Menez J, Echchakir H, Grunenwald D, et al (2002) Tumor-infiltrating CD4+ T lymphocytes express APO2 ligand (APO2L)/TRAIL upon specific stimulation with autologous lung carcinoma cells: role of IFN-alpha on APO2L/TRAIL expression and -mediated cytotoxicity. J Immunol 169: 809–817. 12097384 10.4049/jimmunol.169.2.809

[41] Fanger NA, Maliszewski CR (1999) Schooley K, Griffith TS (1999) Human dendritic cells mediate cellular apoptosis via tumor necrosis factor-related apoptosis-inducing ligand (TRAIL). J Exp Med 190: 1155–1164. 10523613 10.1084/jem.190.8.1155 PMC2195665

[42] Griffith TS, Wiley SR, Kubin MZ, Sedger LM, Maliszewski CR, et al (1999) Monocyte-mediated tumoricidal activity via the tumor necrosis factor-related cytokine, TRAIL. J Exp Med 189: 1343–1354. 10209050 10.1084/jem.189.8.1343 PMC2193036

[43] Chan J, Prado-Lourenco L, Khachigian LM, Bennett MR, Di Bartolo BA, et al (2010) TRAIL promotes VSMC proliferation and neointima formation in a FGF-2-, Sp1 phosphorylation-, and NFkappaB-dependent manner. Circ Res 106: 1061–1071. 20150555 10.1161/CIRCRESAHA.109.206029

